# People’s attitudes towards the agrifood system influence the value of ecosystem services of mountain agroecosystems

**DOI:** 10.1371/journal.pone.0267799

**Published:** 2022-05-04

**Authors:** Enrique Muñoz-Ulecia, Alberto Bernués, Daniel Ondé, Maurizio Ramanzin, Mario Soliño, Enrico Sturaro, Daniel Martín-Collado

**Affiliations:** 1 Unidad de Producción y Sanidad Animal. Centro de Investigación y Tecnología Agroalimentaria de Aragón (CITA), Instituto Agroalimentario de Aragón (IA2) (CITA-Univ. de Zaragoza), Zaragoza, Spain; 2 Department of Psychobiology & Behavioral Sciences Methods, Complutense University of Madrid, Pozuelo de Alarcón, Madrid, Spain; 3 Department of Agronomy, Food, Natural resources, Animals and Environment (DAFNAE), University of Padova, Legnaro, Padova, Italy; 4 Institute of Marine Research, CSIC, Vigo, Spain; 5 Complutense Institute for International Studies (ICEI), Finca Mas Ferré, Edif. A. Campus de Somosaguas, Madrid, Spain; Gebze Teknik Universitesi, TURKEY

## Abstract

Studies covering the social valuation of ecosystem services (ES) are increasingly incorporating people’s attitudes, which allows social heterogeneity to be identified. This is especially relevant in mountain areas, where diverse complex interactions occur among the environment, the socioeconomic system, and a wide variety of farming practices. In this context, we aimed to: (i) identify the attitudinal dimensions that build people views about the agrifood system; and (ii) analyse how these attitudinal dimensions influence the value given to ES delivered by mountain agroecosystems of two European countries. We conducted a survey with a sample of 1008 individuals evenly distributed in the Italian Alps and Spanish Mediterranean mountain areas to collect information on people’s attitudes toward: (i) the economy and the environment; (ii) rural development and agricultural intensification; (iii) food quality, production, and consumption; and (iv) agricultural and environmental policies. The survey included a choice experiment to assess the value that individuals attach to the most relevant ES provided by mountain agroecosystems in these areas (i.e., landscape, biodiversity, quality local products, wildfires prevention and water quality). The results showed four common attitudinal dimensions, namely *Economy over environment*, *Mass-Market distribution reliability*, *Agricultural productivism*, and *Environmentalism and rural lifestyle*. These attitudinal dimensions resulted in six groups of respondents. Most groups positively valued an increase in the delivery of all the analysed ES, which suggests that agricultural policies which aim to promote ES are likely to receive social support in the study areas. However, the differing attitudinal dimensions underlying people’s preferences may result in disagreements about the steps to be taken to achieve the desired increase in ES delivery.

## 1. Introduction

People value things differently, with a very high diversity of individual perceptions of the utility provided by goods and services [1, 2]. This diversity is particularly high in how people perceive the utility derived from ecosystems services (ES) [3]. In this regard, considering people heterogeneity in ES values has been recommended to support inclusive decision making [3], which is frequently addressed by including explanatory variables, such as people’s attitudinal profiles and socio-demographic characteristics [4–6].

In the human-environment interactions field where ES valuation studies are found, research into the relationship between people’s attitudinal profiles and their valuation of ES is increasing. Researchers have focused mostly on studying the influence of attitudes towards the environment on people’s willingness to pay (WTP) or preferences for different ES provision levels [7–9]. These studies have generally found widespread environmental concern (in European Union countries and the United States), and a positive relation between pro-environmental attitudes and high ES values. With agroecosystems, where diverse interactions occur among the environment, socioeconomic systems and farming practices, people’s attitudes towards topics, such as lifestyle, agrifood chains, or what taxes are intended for, among others, may also influence their ES valuation [10, 11]. Therefore, exploring attitudes beyond the degree of environmental concern, including aspects like the agrifood system, the economy-environment relation, the quality and marketing of food products, maintaining the rural lifestyle or different agricultural practices (e.g., organic vs. conventional), may provide a more comprehensive view of society’s perception of ES provided by agroecosystems [12].

Mountain agroecosystems are widely recognised as being highly multifunctional, and supply a variety of cultural, regulating, supporting, and provisioning ES to local inhabitants, visitors, and society in general [12, 13]. However, these agroecosystems are immersed in a continuous process of farming abandonment and intensification in more favourable areas, which impacts ES delivery and raises concerns about their proper management over time [14, 15]. So, identifying social groups according to their attitudes towards the agrifood system and their valuation of the ES delivered by agroecosystems may shed light to design sustainable policies to enhance rural development and food security, while being aware of potential conflicts between different social groups and stakeholders [16–18]. In this regard, [19] explored these aspects and found that people’s valuation of the ES provided by mountain agroecosystems varied between social groups with pro-environmental and agricultural productivism attitudes.

In this context, the purpose of this study was to analyse how people attitudes towards the agrifood system may be modulating their values of the ES provided by European mountain agroecosystems. Specifically, our first aim was to identify the attitudinal dimensions that build people’s views about: (i) the economy and the environment, (ii) rural development and agricultural intensification, (iii) food quality, production, and consumption, and (iv) agricultural and environmental policies. Our second aim was to analyse how these attitudinal dimensions influence the values attached to the ES provided by mountain agroecosystems in two European countries.] followed a standard methodological approach that consisted in including a selection of attitudinal statements in choice models that maximise heterogeneity [4, 19, 20]. However, this method shows marked differences in people’s attitudes due to the selection of those statements that receive the most extreme responses [21]. Moreover, the selection of opinion variables without considering their psychometric properties, might generate endogeneity problems in econometric models [10, 22]. In this study, we follow a more solid alternative to include people’s attitudes in choice experiments, which consists in considering the latent attitudinal dimensions that underlie and shape people’s responses [21, 23].

## 2. Materials and methods

### 2.1. Study areas

Our study builds on the research performed by previous studies [19, 24, 25]. The study areas are located in High Nature Value (HNV) mountain regions of Spain and Italy. In Spain, the ‘Sierra y Cañones de Guara’ Natural Park in the Spanish Pre-Pyrenees is a protected area of 807 km^2^ in northeast Spain. It is a Mediterranean mountainous area characterised by extensive, low-input low-output livestock farming systems that form a highly heterogeneous agricultural landscape [26]. These agroecosystems are recognised for their provision of numerous ES, such as biodiversity conservation, wildfires control, and the supply of quality products linked to the territory [25]. In Italy, the Autonomous Province of Trento in the Italian Alps covers an elevated mountainous area of 6200 km^2^ in northern Italy. This region works mainly in traditional dairy cattle agroecosystems which, similarly to the Spanish case study, provide diverse ES, such as a wide variety of local cheeses, highly valued landscapes and biodiversity conservation [27–29]. However, the combined processes of agriculture intensification and abandonment that have taken place in recent decades in both areas have led to a disruption of the ES that these agroecosystems provide society with [30, 31].

### 2.2. Survey design

The Ethics Committee of the Agrifood Research and Technology Centre of Aragón, Spain, approved the research protocol and questionnaire content (no. CEISH_2021_3). Data anonymity was granted to the participants in the survey, who expressed their oral consent to provide the information contained in the questionnaire. Focus groups were performed in each study area to collect local inhabitants’ opinions about the relation between livestock systems and the environment to identify the ES provided by the livestock agroecosystems under study. Based on these focus groups, a questionnaire was designed and a pilot face-to-face interview was held with 70 respondents to check the questionnaire’s coherence. The final questionnaire included three sections: i) attitudinal statements, ii) ES valuation, and iii) respondents’ socio-economic data (e.g., age, gender, family size, level of education, level of income, relationship and involvement in farming and environmental associations). Interviews were held with 1008 people. In each study area, 102 adult residents in the mountain areas under study (i.e., local population) were interviewed face-to-face in all the rural areas, and 402 adults were interviewed by a professional online panel to represent the general population in the regions where the study areas are located (i.e., Aragon in Spain, Trento Province in Italy). Further details about the questionnaire design and sampling processes are found in [19, 24, 25].

#### 2.2.1. Attitudinal statements

We defined 20 Likert statements on aspects that might play a direct or indirect role for ES provision, which have been previously used by [19]. Statements were grouped into four main topics: (i) economy and environment, (ii) rural development and agricultural intensification, (iii) food quality, production, and consumption, and (iv) agricultural and environmental policies (Table 1). Statements were randomly (using the Microsoft Excel randomization tool) presented to respondents, who were asked to state their level of agreement with each statement on a five-point Likert scale ranging from *1—“Totally disagree”* to *5—“Totally agree”*, with an intermediate option for “*Neutral*”.

**Table 1:** Statements used in the questionnaire and descriptive statistics.

Statements by topic	Mean (SD)	Median	Skewness (Kurtosis)
Economy and environment (EE)			
EE1. We need to change the economic model to integrate better the conservation of the environment	4.2 (0.8)	4.0	-1.3 (2.1)
EE2. Economic growth is more important than preserving nature	2.0 (1.0)	2.0	1.1 (0.6)
EE3. We need to maximize profit obtained from natural resources	3.3 (1.3)	3.0	-0.2 (-1.2)
EE4. We should change our lifestyle and consume less	3.7 (1.1)	4.0	-0.6 (-0.3)
EE5. Climate change is one of the biggest challenges’ we humans face	3.9 (1.0)	4.0	-1.0 (0.6)
Rural development and agricultural intensification (RD)			
RD6. We must invest more in stopping rural depopulation and abandonment	4.4 (0.8)	5.0	-1.5 (2.8)
RD7. When I go to the countryside, I prefer landscapes with no human intervention (e.g., high mountains)	4.2 (0.9)	4.0	-1.0 (0.6)
RD8. If I could choose, I would live in the countryside rather than in a city	3.7 (1.2)	4.0	-0.6 (-0.7)
RD9. Livestock production is always negative for the environment	2.0 (1.0)	2.0	0.9 (0.6)
RD10. Intensive agriculture (industrial) is the best way to solve hunger in the world	2.5 (1.1)	2.0	0.4 (-0.5)
Food quality, production and consumption (FQ)			
FQ11. I normally look for information on how foods are produced and their origin	3.8 (0.9)	4.0	-0.6 (0.2)
FQ12. New technologies in food processing and packaging increase product quality	3.1 (1.1)	3.0	-0.1 (-0.7)
FQ13. Organic, local and seasonal products are good alternatives for fairer and sustainable consumption	4.3 (0.9)	4.0	-1.2 (1.5)
FQ14. Supermarkets offer better guarantee of food quality than traditional shops	2.4 (1.0)	2.0	0.5 (0.0)
FQ15. Supermarkets offer better guarantee of food safety than traditional shops	2.5 (1.0)	2.0	0.3 (-0.4)
Agricultural and environmental policy (AP)			
AP16. Government should reduce the amount of money invested in environmental policies and invest somewhere else	2.0 (1.0)	2.0	1.0 (0.7)
AP17. Agricultural policies and premiums to farmers need to be maintained because agriculture is a strategic sector	3.8 (0.9)	4.0	-0.6 (0.3)
AP18. Agricultural premiums must be given to farmers according to their production level	3.6 (1.1)	4.0	-0.5 (-0.3)
AP19. Farmers in mountain and other less favoured/remote areas should receive higher premiums	3.8 (0.9)	4.0	-0.5 (-0.1)
AP20. Agricultural and environmental policies need better targeting and control	4.2 (0.8)	4.0	-1.2 (2.0)

#### 2.2.2. Ecosystem services valuation

A discrete choice experiment was used to value the most relevant ES provided by the mountain agroecosystems under study, which were identified in the initial pilot interview. The use of choice experiments instead of other valuation methods is based on its higher robustness to analyse several attribute levels [32]. The experiment represented different agriculture policy scenarios, whose implementation would lead to distinct ES provision levels. Both attributes (cultural, supporting, regulating and provisioning ES, and cost) and levels (improvement, maintenance, and decrease) were defined to ensure that they were intuitive for the general public, scientifically accepted, and also appropriate for identifying changes in ES provision [25, 33, 34]. ES were site-specific, which allows people to value their local and known environment. The cultural, supporting and provisioning ES were the same in both areas, namely landscape, biodiversity, and quality local products, respectively. Regulating ES referred to forest wildfires prevention in Spain and water quality in Italy. It was explicitly clear that the cost of each alternative corresponded to the amount of money that each family member older than 18 would have to pay as annual tax. Further details about the choice experiment design are found in [19, 24, 25].

**Fig 1:**
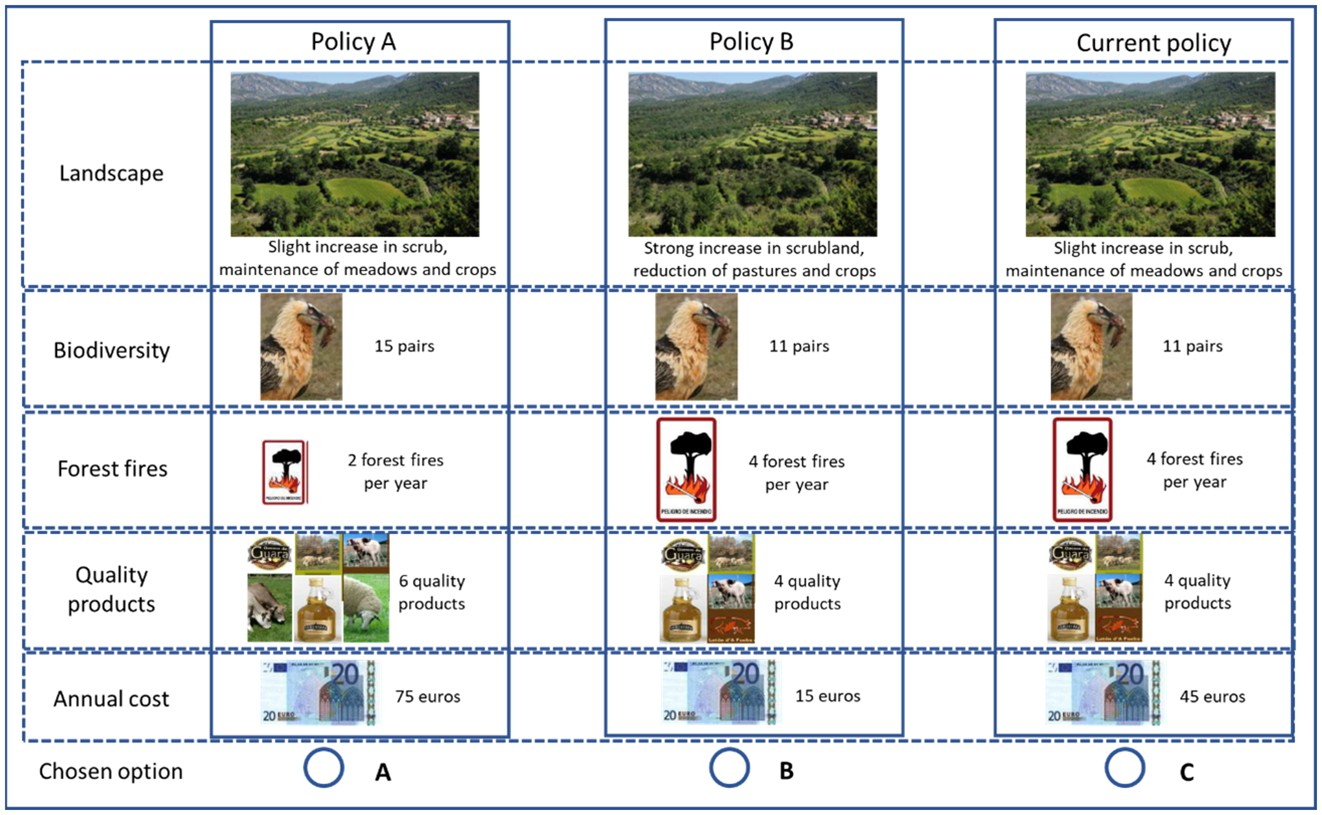
Example of the choice set presented to the respondents in the Spanish ‘Sierra y Cañones de Guara’ Natural Park. From [25]. Policy A, B, and Current policy, refers to ES improvement, decrease, and maintenance, respectively.

During the choice experiments, individuals were asked to choose their preferred alternatives from 30 choice sets divided into six blocks [5]. Each choice set consisted of three alternatives described by attributes (ES and costs of agri-environmental schemes), which took multiple levels according to the scenarios created following an efficient fractional factorial design and orthogonal and balanced combinations for the final design. Choices required the respondents making a trade-off between ES levels and tax costs and, thereby, stating their preferences for the ES levels (Fig 1) with the option to opt-out to avoid forced choices.

### 2.3. Statistical analysis

We firstly performed an Exploratory Factor Analysis (EFA) to reveal the respondents’ attitudinal dimensions (i.e., factors) that underly their attitudes towards: the economy and the environment; rural development and agricultural intensification; food quality, production and consumption; and agricultural and environmental policy. Having determined and described the factors, we included them in a latent-class choice model to explore their influence on people’s ES valuation. Finally, we tested any differences between the latent classes (social groups hereafter) identified in the choice model regarding socioeconomic variables using the Kruskal-Wallis and Chi^2^ tests for numeric and categorical variables, respectively.

#### 2.3.1. Attitudinal dimension analysis

We used the polychoric correlation matrix as the input matrix, which is appropriate when analysing ordinal variables (i.e., attitudinal statements; see [35]). We employed the Psych package of R [36] and the estimator Unweighted Least Squares (ULS). The resulting factors identified groups of related scores to the attitudinal statements, which allowed us to look more closely at the respondents’ inner attitudinal structure by determining attitudinal dimensions. The Parallel Analysis (PA) was followed to identify the optimum latent structure of data and, thus, the adequate number of factors. To test the model’s fit, we checked different indicators (e.g., Root Mean Square Error of Approximation (RMSEA), Non-Normed Fit Index (NNFI), Root Mean Square Residuals (RMSR), and residuals).

#### 2.3.2. Choice modelling

We used a latent-class choice model [37] with random parameters to estimate each individual’s utility that derived from a particular ecosystem service with the Latent GOLD^®^ software (v. 5.1). This model type allows different social groups to be isolated using the attitudinal dimensions identified in the EFA, which enables the unobserved preference heterogeneity among the respondents from each social group to be considered. For each social group, we examined the relation between individuals’ preferred policy choices (dependent variable) and the levels of the attributes in the alternatives they chose, i.e., landscape maintenance, biodiversity preservation, maintaining water quality/preserving forest fires, provision of high-quality local food products and annual cost (independent variables). The effect of the attributes on choice probability was evidenced by parameter estimates. The sign of a parameter value showed the extent to which the presence of an attribute in each scenario influenced the probability of choosing it. A further description of the latent-class choice model specifications is found in the S1 Appendix.

## 3. Results

### 3.1. Psychographic analysis—Respondents’ attitudinal dimensions

The EFA gave similar results in both study areas, with the best solution suggested by the PA. It consisted in four factors with adequate fit indices values (Spain: RMSEA = 0.043, NNFI = 0.903, RMSR = 0.031 (< 0.44 Kelley criterion), 4.6% of the residuals > 0.05; Italy: RMSEA = 0.053, NNFI = 0.899, RMSR = 0.032 (< 0.44 Kelley criterion), 5.1% of the residuals > 0.05).

An initial analysis, which considered the pooled sample indicated that statement AP17 (Table 1) was problematic because it showed salient factor loadings on more than one factor. This was why, we removed statement AP17 and re-analysed the dataset. The best solution of the EFA, suggested by the PA, was four factors. The fit indices values were RMSEA = 0.049, NNFI = 0.900, and RMSR = 0.029 (lower value than Kelley criterion 0.032). Only 4.8% of the residuals values were > 0.05. Table 2 shows the configuration matrix of the factor loading on the 19 statements final scale.

**Table 2:** Configuration matrix of the 19 statements factor loadings for attitudinal factors (oblique rotation).

Statements*	Factor 1 *Economy above environment*	Factor 2 *Mass-Market distribution reliability*	Factor 3 *Agriculture productivism*	Factor 4 *Environmentalism and rural lifestyle*
EE2. Economic growth vs nature	**0.396**	0.219	0.211	-0.129
RD9. Livestock always has impact	**0.525**	0.179	-0.161	0.029
AP16. Reduce environmental policies	**0.569**	0.061	0.137	-0.094
FQ12. New techs increase quality	-0.043	**0.366**	0.302	0.011
FQ14. Supermarkets guarantee quality	-0.003	**0.716**	-0.013	-0.033
FQ15. Supermarkets guarantee safety	0.087	**0.753**	-0.001	0.015
EE3. Maximize profit	0.036	-0.040	**0.664**	0.047
RD10. Intensive agric. can solve hunger	0.089	0.294	**0.452**	-0.094
AP18. Premiums coupled to production	-0.029	0.073	**0.400**	0.252
EE1. Change economic model	-0.104	0.078	-0.170	**0.591**
EE4. Change lifestyle	0.184	0.013	-0.271	**0.450**
EE5. Climate change concern	-0.195	0.189	-0.060	**0.348**
RD6. Invest to stop rural depopulation	-0.182	-0.011	0.176	**0.574**
RD7. Non-anthropic landscapes	0.013	-0.020	0.014	**0.465**
RD8. Prefer living in the countryside	0.170	-0.143	0.081	**0.429**
FQ11. Concern about foods origin	0.126	-0.099	0.050	**0.559**
FQ13. Support organic local products	0.043	-0.096	-0.007	**0.672**
AP19. Higher support in remote areas	-0.143	0.077	0.193	**0.375**
AP20. Better control of green policies	-0.122	0.076	0.063	**0.597**

*Statements full description is presented in Table 1. Bold letters refer to the statements that compound each factor.

These four factors represented the attitudinal dimensions that we hypothesised would influence or help to explain the respondents’ ES valuation, and can be described as follows:

- Factor 1 –*Economy over environment*: it represented attitudes towards the generation of economic wealth over nature, acceptance of the livestock impact on the environment, and lack of interest of environmental policies.
- Factor 2 –*Mass-Market distribution reliability*: it represented attitudes towards new technologies for product transformation and large supermarkets as better guarantors of product quality and safety than traditional stores.
- Factor 3 –*Agricultural productivism*: it grouped attitudes towards maximising economic benefits from natural resources, intensive agriculture advantages and productivity-oriented premiums.
- Factor 4 –*Environmentalism and rural lifestyle*: it was formed by half the presented statements, which gathered all the attitudes related to a socio-environmentally concerned way of life and towards rural life.

Some factors were correlated to a limited extent: *Economy over environment* correlated positively with *Mass-Market distribution reliability* (0.37) and negatively with *Environmentalism and rural lifestyle* (-0.32). *Mass-Market distribution reliability* correlated positively with *Agriculture productivism* (0.25) and negatively with *Environmentalism and rural lifestyle* (-0.16). All the other correlations between factors came close to zero.

### 3.2. Choice experiment—Respondents’ ecosystem services valuation

**Fig 2:**
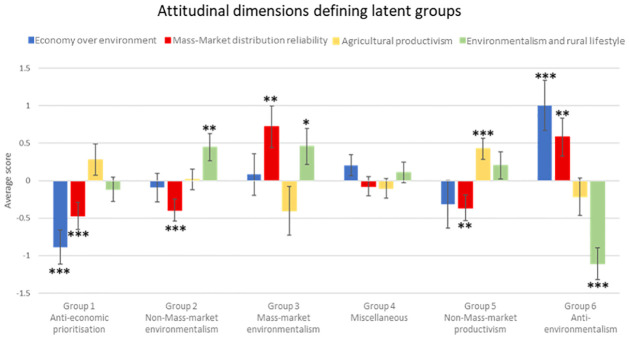
Different social groups formed in the latent-class model from the attitudinal dimensions. Scores indicate the contribution of each attitudinal factor to the different latent social groups. Black stars refer to trends (90%, 1 star) and significant attitudes defining social groups (95% and 99% for 2 and 3 stars, respectively). For more detailed statistical results, see A1 Table in S1 Appendix.

**Fig 3:**
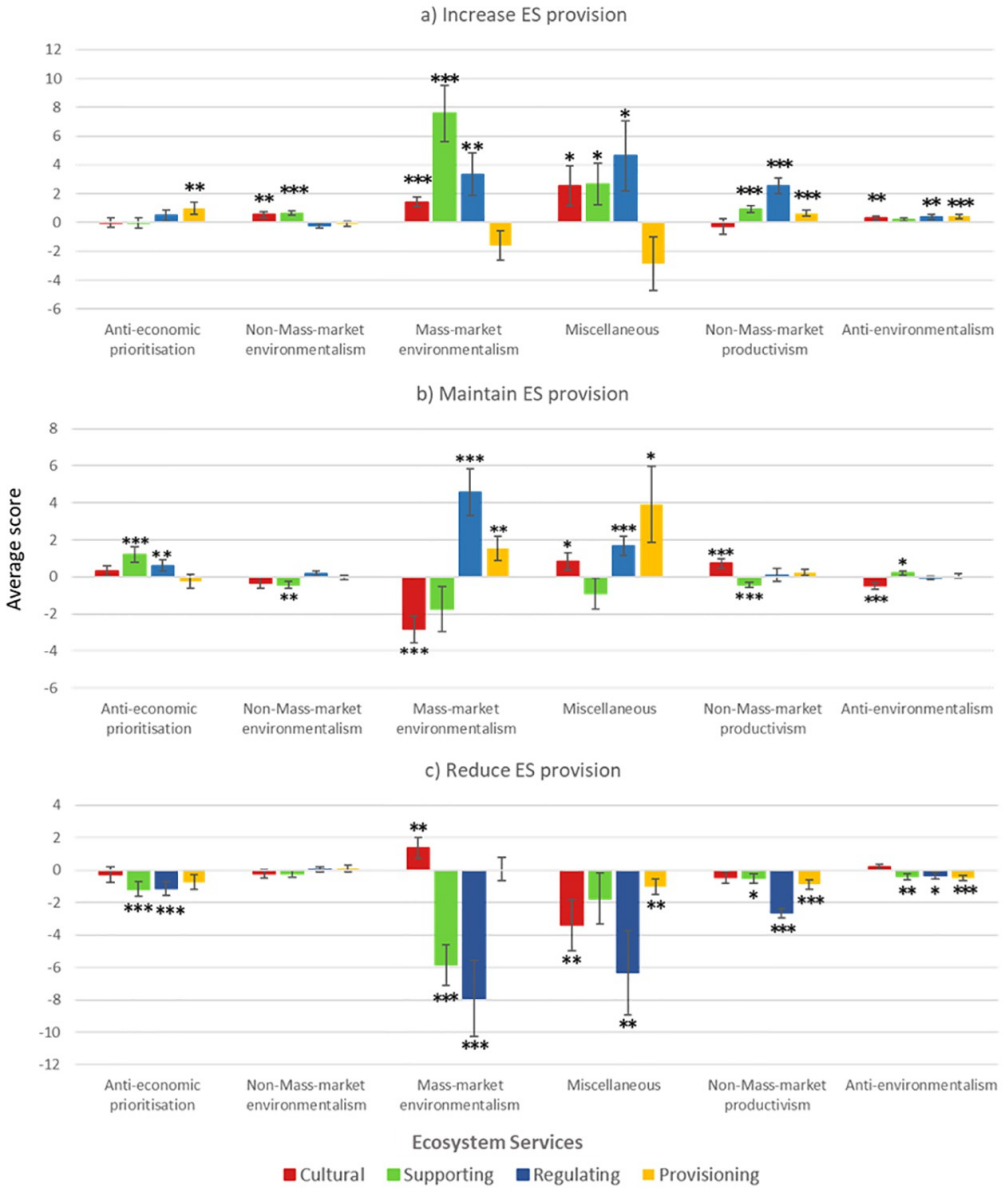
Respondents’ ES valuation for the different latent social groups in the different scenarios. Fig 3a–3c, represent how different social groups valued ES attributes in various scenarios. A more detailed description of scenarios is provided in [24, 25]. Black stars refer to trends (90%, 1 star) and significant scores (95% and 99% for 2 and 3 stars, respectively). Deviation bars denote standard error. For more detailed statistical results, see A2 Table in S1 Appendix.

The model that showed the best fit while allowing a comprehensive explanation resulted in six social groups and had a McFadden pseudo R^2^ of 0.57. Figs 2 and 3 summarise the latent-class model results. Fig 2 describes the latent groups according to the attitudinal dimensions, while Fig 3 shows the value given to ES provided by HNV agroecosystems across social groups. Full model outputs are provided in the S1 Appendix (A1 and A2 Tables in S1 Appendix).

- Group 1 –*Anti-economic prioritisation* (14.6% of the responses) grouped attitudes strongly opposed *Economy over environment* and opposed to *Mass-Market distribution reliability*. This social group showed support to the current level and were against the deterioration of supporting and regulating ES, while also positively valuing an improvement in provisioning ES. They did not show a clear position about cultural ES.
- Group 2 –*Non-Mass-market environmentalism* (21.9%) grouped attitudes in favour of *Environmentalism and rural lifestyle* and opposed to *Mass-Market distribution reliability*. They were dissatisfied with the current level of supporting ES and showed support for improving cultural and supporting ES. They did not show a clear position about regulating and provisioning ES.
- Group 3 –*Mass-market environmentalism* (7.0%) grouped attitudes strongly in favour of *Mass-Market distribution reliability* and slightly in favour of *Environmentalism and rural lifestyle*. This social group was the only one to give positive values at the same time to the deterioration and improvement of one ES (namely, cultural ES), while negatively valuing *status quo*. They strongly supported improvement in supporting and regulating ES, but were against their deterioration, while positively valuing the current provisioning ES level.
- Group 4 –*Miscellaneous* (16.7%) presented no dominant attitudinal dimension. This social group was strongly against the deterioration of cultural and regulating ES, and against the deterioration of provisioning ES. They tended to support improvement in cultural, supporting and regulating ES.
- Group 5 –*Non-Mass-market productivism* (25.9%) grouped responses in favour of *Agricultural productivism* and opposed to *Mass-Market distribution reliability*. This social group supported improvement in, but against, the deterioration of supporting, regulating, and provisioning ES. For cultural ES, the current level was the most valued.
- Group 6 –*Anti-environmentalism* (13.9%) grouped attitudes strongly in favour of *Economic over environment* and *Mass-Market distribution reliability*, but strongly opposed to *Environmentalism and rural lifestyle*. These respondents showed support for improvement in, and were against, the deterioration of regulating and provisioning ES. They were dissatisfied with the current level of cultural ES and favoured their improvement. They negatively valued deterioration of supporting ES in favour of the current level.

Note that *Non-Mass-market environmentalism* and *Mass-market environmentalism* (covering one third of the sample) assigned significant positive values to cost estimates (p<0.05 and p<0.01, respectively). This means that these participants did not make the expected trade-off between ES attributes and their associated costs (A2 Table in S1 Appendix). *Non-Mass-market productivism* and *Anti-environmentalism* (around 40% of the sample) attempted to maximise costs (p<0.01 and p<0.05, respectively), while the other social groups (*Anti-economic prioritisation* and *Miscellaneous*) did not offer any significant outputs for this issue.

The analysis of the socio-economic data collected in the third part of the questionnaire (A3 Table in S1 Appendix) showed that *Non-Mass-market productivism* and *Anti-environmentalism* had the lowest income of all the social groups. Significant differences were also found in the respondents’ relationship with the farming sector, being *Environmentalism*, both *Non-Mass-market* and *Mass-market*, who had the lowest proportion. However, no differences were found for the respondents’ age, gender, level of education, relationship with the environment, farming activity and involvement in environmental associations.

## 4. Discussion

The consideration of people’s attitudes in sociocultural valuation studies strongly influence on ES perception [3]. Most studies have focused on environmental attitudes [e.g., 38, 39], leaving attitudes towards other issues largely unexplored. This research gap is particularly for agroecosystems where attitudes towards the agrifood system and the socioeconomic system at large may have great influence on people’s ES valuation [19]. Our study contributes to bridge this gap by exploring a set of attitudes that goes beyond the human-environment relationship using sound psychometric and choice modelling statistical tools. We specifically explored attitudes towards the economy-environment relation, agriculture, food production and consumption, and rural development.

Our results showed that, despite human-environment attitudes and the valuation of ES delivered by agroecosystems usually being context-dependent [40–42], some common attitudinal dimensions shape people’s ES valuation in the Italian Alps and the Spanish Mediterranean mountains. We identified four attitudinal dimensions (namely *Economy over environment*, *Mass-Market distribution reliability*, *Agricultural productivism*, and *Environmentalism and rural lifestyle*) that strongly influenced the respondents’ valuation of the ES provided by mountain agroecosystems.

### 4.1. Attitudinal dimensions

The four attitudinal dimensions revealed different perceptions of the agrifood system, with interrelated aspects related associated with the balance among economy and environment, food production and consumption, agriculture, and rural development.

#### 4.1.1. Economy over environment

This attitudinal dimension shares an epistemological basis with the prevailing socioeconomic paradigm [43], characterised by considering economic growth to be a feasible and advisable social objective [44]. This linkage is sustained by the belief in continuous economic growth as the primary goal, which overrides environment preservation [45] and sustainability [46, 47].

#### 4.1.2. Mass-market distribution reliability

Food processing, quality standards and distribution channels have largely changed in the last few decades [48, 49] by increasing market concentration, with a significant increase in supermarkets at the expense of traditional shops [50, 51]. This attitudinal dimension refers to people’s perception of reliability on mass-market distribution channels as a guarantee of food product quality and safety. Our results showed that this attitudinal dimension has not always confronted pro-environmental positions, as pointed out in other studies [52].

#### 4.1.3. Agricultural productivism

This attitudinal dimension is directly related to the well-known paradigm of productivism, which can be associated with the “Green Revolution”. Agricultural productivism has been considered the paradigmatic strategy to feed the world for a long time [53]. Nowadays, most international agriculture institutions’ strategies recognise agriculture as a multifunctional activity [54, 55]. Our results showed that the productivism paradigm is still established on a large share of the studied population, despite the volume of food production in European countries amply meeting requirements, and problems associated with mountain areas’ agriculture being widely recognised as others (abandonment, landscape degradation, etc.) than maximising production [40].

#### 4.1.4. Environmentalism and rural lifestyle

This attitudinal dimension places attitudes related to both environmental concerns and support of rural lifestyle together. To some extent, it relates the idea of returning to the countryside being the solution to the environmental problems that derive from the current urban lifestyle, which has been profoundly studied by Dunlap and colleagues [46, 56].

### 4.2. Social groups and ecosystem services values

The four identified attitudinal dimensions generated six social groups with a diversity of perspectives towards agriculture, rural lifestyle, product quality and safety, and economy-environment interaction, which resulted in differing ES valuations. However, we observed that, save a few exceptions, all the social groups supported greater ES delivery. This result is consistent with several studies that have demonstrated a general societal willingness to improve ES delivery (even at the expense of paying more taxes) [24, 25, 57] and widespread serious environmental concerns [58–60]. We also found that the respondents’ socioeconomic characteristics (e.g., age, gender and level of education) were not significantly different in the identified social groups, which other studies have also encountered [6]. Below we discuss the different ES valuation across the identified social groups.

#### 4.2.1. Cultural ecosystem services

These non-marketable and non-extractive public services have been increasingly studied in mountain areas in recent years [61, 62]. As cultural ES valuations are inherently subjective and individual-dependent [63], their appropriate management is a source of conflict between different stakeholders and social groups [64–66]. Indeed we found that people from contrasting social groups (*Non-Mass-Market Environmentalism*, *Mass-Market Environmentalism* and *Anti-Environmentalism)* positively valued an increase in cultural ES delivery but would likely support alternative ways of doing so. For example, we observed that, although the people in *Mass-Market environmentalism* social group did not appreciate the current landscape, they supported both a richer mosaic resulting from more agriculture and a re-naturalised landscape resulting from further agriculture abandonment.

#### 4.2.2. Supporting ecosystem services

Supporting ES are often identified with biodiversity preservation, which is considered a key driver for the delivery of many other ES [67]. Our results showed that around 70% of the responses supported increased biodiversity, and the *Mass-Market environmentalism* was the social group with the highest scores. Interestingly, the people in the *Non-Mass-market productivism* social group supported increased biodiversity despite agriculture intensification and industrialisation having been identified as drivers of ES disruption and biodiversity loss [68, 69].

#### 4.2.3. Regulating ecosystem services

These ES are related to ecosystem processes and functions, whose valuation is difficult because the benefits they provide society with are not direct and are, thus, difficult to identify [70]. However, regulating ES are increasingly recognised by society because their role is essential for preventing hazards and maintaining ecosystems’ health [71]. This may explain the general positive attitude towards improving regulating ES in our study. Indeed, most social groups gave high scores against decreasing the regulation of ES delivery.

#### 4.2.4. Provisioning ecosystem services

These marketable and tangible services are private goods that are well recognised by society for being the basis of market trade [72]. Their maximisation usually requires a trade-off with the provision of other services because farmers have to decide between agricultural productivity and pro-environmental practices [73, 74]. We observed that only those groups with a positive or undefined attitude towards *Environmentalism and rural lifestyle* supported an increase in the provision of local quality products.

### 4.3. Considerations for stated preferences studies

Notwithstanding choice experiments are commonly used for monetary ES valuations [75], we identified that one-third of the respondents, specifically those with pro-*Environment and rural lifestyle* (i.e., *Non-Mass-market environmentalism* and *Mass-market environmentalism* attitudes), did not show the expected trade-off between levels of attributes and their associated costs. The inclusion of people’s attitudes in the choice model revealed (an otherwise hidden fact) that a significant part of the participants did not react to the costs attribute of the selected alternative, which does not allow the WTP to be calculated for these groups of respondents. For environmentally concerned people, the prices presented in the experiment were not as important as other characteristics when determining the utility of ES. This finding suggests that the WTP calculation might be over-estimated by strong pro-environmental attitudes [20]. However, we should acknowledge that the cost associated with different choice alternatives could be too low for some respondents despite the pilot survey experience. This involves a handicap to implement or select choice alternatives that mean the same to everyone. These results confirm the existing literature, and highlight that methods very much shape valuation outcomes [76]. Previous studies that have applied a mixed logit modelling approach have pointed out the presence of high heterogeneity on several dimensions [24, 77]. Our result encourages the inclusion of people’s attitudes in choice experiments to account for such heterogeneity, and the use of complementary non-monetary approaches to assess the value of ES [75, 78].

### 4.4. Limitations

We should highlight some limitations in our study that join to those previously acknowledged in [38, 39]. We focused on the most relevant ES identified during participatory workshops held with local stakeholders. Consequently, our research did not cover all the ES provided by the studied agroecosystems. We should note that wildfire prevention and improvement of water quality have been highly recognised and valued by society [24, 77]. This raises questions as to whether less socially recognised ES would lead to different results.

Regarding the link between attitudes and behaviour, despite it is commonly assumed that people behave according to their values, attitudes have been found to have a varying impact on real behaviour, which results from the complex interaction between different external (e.g., economic and cultural) and internal factors (e.g., motivation, awareness, values, locus of control, priorities) [79–81]. Moreover, some degree of hypothetical bias underlying choice design and attribute non-attendance (as recognised above for the cost attribute) might also be present in the study [82, 83], so future research should look closely at these relevant issues for economic valuation applications.

### 4.5. Insights for policy planning

Social and scientific claims for designing transformative policies to face the sustainability challenge are increasing [84, 85]. However, large-scale transitions require coordinated plans and society’s commitment at different levels and scales [86–88]. As society integrates heterogeneous social groups with contrasting values and goals, the identification of social heterogeneity can help to design strategies that modify those values and engage hesitant societal sectors in more sustainable behaviours [89, 90].

Our study shows some common attitudinal dimensions related to the agrifood system across study regions that, if confirmed in other European mountain areas, may help to understand the social acceptance of alternatives in agricultural and nature conservation policies. This study also reveals that society might be highly segmented in different social groups regarding these attitudinal dimensions, which can lead to a double reading: on the one hand, despite this segmentation, we found widespread support to improve the delivery of some ES across all social groups (e.g., the general preference for higher biodiversity levels across social groups may facilitate the implementation and success of biodiversity policies, such as the European Biodiversity Strategy 2030) [91]; on the other hand, since a generalised consensus on the desired goal (improve ES delivery) does not always imply agreements about the ways and means to achieve it, conflicts are likely to emerge. In the end, these conflicts may determine the success or failure of policies, as highlighted by other authors [89, 92].

## 5. Conclusions

This study contributes to understand people’s attitudes towards central topics in the agrifood systems-environment debate, including the livestock environmental impact, the quality and marketing of food products, and rural development. This research work establishes four attitudinal dimensions: *Economy over environment*, *Mass-Market distribution reliability*, *Agricultural productivism*, and *Environmentalism and rural lifestyle*. These attitudinal dimensions influence the values that people attach to the key ES provided by mountain agroecosystems, which allowed us to identify diverse social groups. The preference for increasing ES delivery in most groups highlights the social demand for policies that aim to increase the delivery of ES in mountains. However, the differing attitudinal dimensions that underly people’s preferences may result in disagreement and conflict about the specific policy measures to be implemented.

## Supporting information

S1 Appendix (DOCX)
