# Supplementary material for: People’s attitudes towards the agrifood system influence the value of ecosystem services of mountain agroecosystems
Source: PLoS One. 2022 May 4;17(5):e0267799. doi: 10.1371/journal.pone.0267799 (PMC9067659; doi:10.1371/journal.pone.0267799)
Supplement: S1 Appendix — (DOCX) [file pone.0267799.s001.docx]

**Appendix**

**Model description. Latent class choice model with random parameters description.**

Individuals *i* choose an alternative from a set of choices in *t* cards (*t* = 1, 2, …, T). The individual utility *i* from alternative *j* in the choice card *t* is: U*_ijt_* = β*_i_* *x_ijt_* + γ*_i_* *c_ijt_* + ε*_i_*

Where *x_ijt_* is the vector of the ES attributes for each alternative *j*, c*_ijt_* is the monetary attribute, β*_i_* and γ*_i_* are their respective estimated coefficients, and ε*_i_* is an unobservable error term.

A latent class model was used to analyse data (37). The model considers several classes (*x*) for the probability that individual *i*’s choice *j* at card *t,* given an attribute vector$z_{\mathrm{it}}^{\mathrm{att}}$ (*x_ijt_*, c*_ijt_*), and the random parameters denoted by *F_i_* are assumed to be standard normally distributed and mutually independent (93). In a conditional logit model, the response probability is:

$P(y_{it}=j|x,z_{it}^{att},F_{i})=\frac{\exp(\eta_{j|x,z_{it},F_{i}})}{\sum_{j'=1}^{J} \exp(\eta_{j|x,z_{it},F_{i}})}$ (1)

Where $\eta_{j|x,z_{it}}$ is the systematic component in the utility of alternative *j* at card *t* given that individual *i* belongs to latent class *x*. Therefore, if we consider a latent class choice model with random parameters, the probability density is:

$P(y_{i}|z_{i})=\sum_{x=1}^{K} \int_{F_{i}} \int(F_{i})P(x|z_{i})(F_{i})\prod_{t=1}^{T_{i}} P(y_{it}|x,z_{it}^{att},F_{i})dF_{i}$ (2)

Finally, the marginal willingness to pay for an attribute *k* can be estimated as the negative ratio between the coefficient estimated for attribute *k* and the cost coefficient.

Table A1. Latent-class model results representing the six found groups (including standard error and z-value) and the attitudinal dimensions defining each group.

| Intercept | Group 1  Anti-economic prioritisation | s.e. | z-value | Group 2 Non-Mass-market environmentalism | s.e. | z-value | Group 3 Mass-market environmentalism | s.e. | z-value | Group 4 Miscellaneous | s.e. | z-value | Group 5 Non-Mass-market productivism | s.e. | z-value | Group 6 Anti-environmentalism | s.e. | z-value | Wald | p-value |
| --- | --- | --- | --- | --- | --- | --- | --- | --- | --- | --- | --- | --- | --- | --- | --- | --- | --- | --- | --- | --- |
|  | 0.05 | 0.25 | 0.20 | 0.64 | 0.20 | 3.28 | -0.78 | 0.36 | -2.18 | 0.45 | 0.17 | 2.68 | 0.82 | 0.23 | 3.63 | -1.19 | 0.45 | -2.67 | 26.43 | 0.00 |
| R²^+^ | 0.37 |  |  | 0.46 |  |  | 0.84 |  |  | 0.75 |  |  | 0.45 |  |  | 0.18 |  |  |  |  |
| Covariates |  |  |  |  |  |  |  |  |  |  |  |  |  |  |  |  |  |  |  |  |
| Factor 1. Economy above environment | | | | | | | | | | | | | | | | | | | | |
|  | -0.89*** | 0.23 | -3.89 | -0.09 | 0.19 | -0.48 | 0.08 | 0.28 | 0.29 | 0.21 | 0.14 | 1.48 | -0.31 | 0.32 | -0.99 | 1.01*** | 0.34 | 3.00 | 19.42 | 0.00 |
| Factor 2. Mass-Market distribution reliability | | | | | | | | | | | | | | | | | | | | |
|  | -0.47*** | 0.18 | -2.60 | -0.39*** | 0.15 | -2.64 | 0.72** | 0.28 | 2.57 | -0.08 | 0.13 | -0.60 | -0.36** | 0.17 | -2.11 | 0.58** | 0.25 | 2.30 | 15.26 | 0.01 |
| Factor 3. Agricultural productivism | | | | | | | | | | | | | | | | | | | | |
|  | 0.28 | 0.21 | 1.36 | 0.01 | 0.14 | 0.10 | -0.40 | 0.33 | -1.23 | -0.10 | 0.13 | -0.77 | 0.43*** | 0.14 | 3.08 | -0.22 | 0.25 | -0.87 | 12.67 | 0.03 |
| Factor 4. Environmentalism and rural lifestyle | | | | | | | | | | | | | | | | | | | | |
|  | -0.11 | 0.16 | -0.69 | 0.45** | 0.18 | 2.46 | 0.46* | 0.24 | 1.90 | 0.11 | 0.14 | 0.79 | 0.20 | 0.18 | 1.14 | -1.10*** | 0.21 | -5.20 | 28.51 | 0.00 |

Black stars refer to trends (90%, 1 star) and significant attitudinal dimensions defining groups (95% and 99% for 2 and 3 stars, respectively). ^+^McFadden pseudo R² = 0.57; Entropy R^2^: 0.65

Table A2. Choice model results. It shows scores, standard error and z-values.

| Attributes* | Group 1  Anti-economic prioritisation | s.e. | z-value | Group 2 Non-Mass-market environmentalism | s.e. | z-value | Group 3 Mass-market environmentalism | s.e. | z-value | Group 4 Miscellaneous | s.e. | z-value | Group 5 Non-Mass-market productivism | s.e. | z-value | Group 6 Anti-environmentalism | s.e. | z-value |
| --- | --- | --- | --- | --- | --- | --- | --- | --- | --- | --- | --- | --- | --- | --- | --- | --- | --- | --- |
| Cultural ES (Landscape) | | | | | | | | | | | | | | | | | | |
| Reduce | -0.30 | 0.48 | -0.62 | -0.24 | 0.27 | -0.88 | 1.39** | 0.64 | 2.16 | -3.40 | 1.56 | -2.19 | -0.45 | 0.39 | -1.14 | 0.19 | 0.15 | 1.28 |
| Maintain | 0.32 | 0.30 | 1.06 | -0.31 | 0.30 | -1.02 | -2.82*** | 0.72 | -3.94 | 0.84* | 0.48 | 1.76 | 0.72*** | 0.24 | 2.95 | -0.49*** | 0.15 | -3.25 |
| Increase | -0.02 | 0.33 | -0.06 | 0.55** | 0.22 | 2.48 | 1.43*** | 0.35 | 4.05 | 2.56* | 1.39 | 1.85 | -0.27 | 0.52 | -0.53 | 0.30** | 0.14 | 2.24 |
| Supporting ES (Biodiversity) | | | | | | | | | | | | | | | | | | |
| Reduce | -1.17*** | 0.44 | -2.66 | -0.23 | 0.21 | -1.07 | -5.85*** | 1.26 | -4.65 | -1.76 | 1.58 | -1.12 | -0.51* | 0.30 | -1.71 | -0.40** | 0.18 | -2.27 |
| Maintain | 1.20*** | 0.41 | 2.90 | -0.43** | 0.19 | -2.21 | -1.73 | 1.21 | -1.43 | -0.90 | 0.85 | -1.06 | -0.43*** | 0.15 | -2.81 | 0.21 | 0.12 | 1.70 |
| Increase | -0.03 | 0.34 | -0.10 | 0.66*** | 0.16 | 4.07 | 7.58*** | 1.97 | 3.85 | 2.66* | 1.42 | 1.88 | 0.94*** | 0.24 | 3.87 | 0.20 | 0.14 | 1.38 |
| Regulating ES (Wildfires and Water quality) | | | | | | | | | | | | | | | | | | |
| Reduce | -1.11*** | 0.43 | -2.58 | 0.04 | 0.17 | 0.22 | -7.92*** | 2.33 | -3.40 | -6.32** | 2.59 | -2.44 | -2.65*** | 0.31 | -8.52 | -0.35* | 0.21 | -1.67 |
| Maintain | 0.62** | 0.29 | 2.16 | 0.18 | 0.13 | 1.40 | 4.57*** | 1.26 | 3.62 | 1.69*** | 0.51 | 3.34 | 0.10 | 0.36 | 0.28 | -0.05 | 0.10 | -0.53 |
| Increase | 0.50 | 0.38 | 1.32 | -0.21 | 0.17 | -1.27 | 3.35** | 1.46 | 2.29 | 4.63* | 2.44 | 1.90 | 2.55*** | 0.53 | 4.79 | 0.41** | 0.18 | 2.27 |
| Provisioning ES (Quality Products) | | | | | | | | | | | | | | | | | | |
| Reduce | -0.75 | 0.46 | -1.63 | 0.11 | 0.21 | 0.56 | 0.07 | 0.73 | 0.09 | -1.03** | 0.47 | -2.17 | -0.89*** | 0.28 | -3.21 | -0.49*** | 0.18 | -2.68 |
| Maintain | -0.23 | 0.37 | -0.64 | -0.03 | 0.13 | -0.25 | 1.54** | 0.64 | 2.40 | 3.91* | 2.06 | 1.90 | 0.25 | 0.17 | 1.47 | 0.06 | 0.13 | 0.45 |
| Increase | 0.98** | 0.44 | 2.25 | -0.08 | 0.17 | -0.47 | -1.61 | 1.03 | -1.56 | -2.88 | 1.87 | -1.54 | 0.64*** | 0.21 | 3.09 | 0.43*** | 0.14 | 3.07 |
| Price | | | | | | | | | | | | | | | | | | |
|  | -0.00 | 0.03 | -0.13 | 0.11*** | 0.01 | 7.85 | 0.24** | 0.10 | 2.46 | -0.08 | 0.07 | -1.14 | -0.06*** | 0.01 | -4.81 | -0.03** | 0.01 | -2.48 |

Black stars refer to trends (90%, 1 star) and significant values for ES attributes (95% and 99% for 2 and 3 stars, respectively). Different attributes denoted the ES delivery level, from the current level to an improvement or reduction in the ES delivered level.

Table A3. Respondents’ socioeconomic characteristics.

| Socio-economic variables | | Group 1  Anti-economic prioritisation (14.6%) | Group 2 Non-Mass-market environmentalism (21.9%) | Group 3 Mass-market environmentalism (7.0%) | Group 4 Miscellaneous (16.7%) | Group 5 Non-Mass-market productivism (25.9%) | Group 6 Anti-environmentalism (13.9%) | Significance (P-value) |
| --- | --- | --- | --- | --- | --- | --- | --- | --- |
| Age (year) | | 41.59 | 42.84 | 39.37 | 42.25 | 41.90 | 42.32 | n.s. |
| Sex (%) | Female | 48.91 | 42.27 | 45.12 | 50.78 | 48.00 | 51.59 | n.s. |
|  | Male | 51.09 | 57.73 | 54.88 | 49.22 | 52.00 | 48.41 |  |
| Family size (nº of people) | | 3.00ab | 2.95ab | 3.09ab | 3.11ab | 3.22a | 2.81b | n.s. |
| Level of education (%) | High | 45.99 | 39.09 | 34.15 | 36.27 | 34.00 | 27.20 | n.s. |
|  | Mid | 8.76 | 10.45 | 7.32 | 15.03 | 11.60 | 16.80 |  |
|  | Low | 45.26 | 50.45 | 58.54 | 48.70 | 54.40 | 56.00 |  |
| Level of income (%) | High | 17.95 | 16.77 | 17.14 | 15.09 | 13.99 | 6.38 |  |
|  | Mid-High | 43.59 | 41.32 | 44.29 | 47.17 | 54.92 | 52.13 |  |
|  | Mid-Low | 35.04 | 38.32 | 37.14 | 33.96 | 23.32 | 35.11 | 0.046 |
|  | Low | 3.42 | 3.59 | 1.43 | 3.77 | 7.77 | 6.38 |  |
| Relation with the environment (%) | | 15.33 | 17.81 | 6.17 | 16.06 | 12.40 | 20.80 | n.s. |
| Relation with farming (%) | | 14.71 | 7.31 | 6.17 | 12.95 | 11.34 | 16.80 | 0.046 |
| Farming activity (%) | | 25.55 | 19.55 | 19.75 | 20.73 | 27.42 | 20.63 | n.s. |
| Environmental association (%) | | 9.49 | 11.42 | 12.35 | 6.77 | 4.84 | 6.35 | n.s. |

Kruskal-Wallis (numerical variables) and Chi^2^ (categorical variables) tests were used to compare differences between groups. Level of education represents the percentage of respondents in each category: Low (elementary school), Mid (high school) and High (university). Level of income indicates the percentage of respondents in each category: Low (<700€/month), Mid-low (700–1,500€/month), Mid-high (1,501–3,000€/month) and High (>3,000€/month). Relation with the environment and farming represents the percentage of the respondents who perform an activity related to the environment or agriculture, while Farming activity and Environmental association represent those respondents who are directly involved in farming or in an environmental association.
